# Advanced “Green” Prebiotic Composite of Bacterial Cellulose/Pullulan Based on Synthetic Biology-Powered Microbial Coculture Strategy

**DOI:** 10.3390/polym14153224

**Published:** 2022-08-08

**Authors:** Sirina Zhantlessova, Irina Savitskaya, Aida Kistaubayeva, Ludmila Ignatova, Aizhan Talipova, Alexander Pogrebnjak, Ilya Digel

**Affiliations:** 1 Department of Biotechnology, Al-Farabi Kazakh National University, 71 Al-Farabi Avenue, Almaty 050040, Kazakhstan; 2 Department of Nanoelectronics and Surface Modification, Sumy State University, Ryms’koho-Korsakova St. 2, 40000 Sumy, Ukraine; 3 Institute for Bioengineering, Aachen University of Applied Sciences, Heinrich-Mußmann-Straße 1, 52428 Jülich, Germany

**Keywords:** bacterial cellulose, prebiotic, exopolysaccharides, pullulan, coculture, *Komagataeibacter xylinus*, *Aureobasidium pullulans*

## Abstract

Bacterial cellulose (BC) is a biopolymer produced by different microorganisms, but in biotechnological practice, *Komagataeibacter xylinus* is used. The micro- and nanofibrillar structure of BC, which forms many different-sized pores, creates prerequisites for the introduction of other polymers into it, including those synthesized by other microorganisms. The study aims to develop a cocultivation system of BC and prebiotic producers to obtain BC-based composite material with prebiotic activity. In this study, pullulan (PUL) was found to stimulate the growth of the probiotic strain *Lactobacillus rhamnosus* GG better than the other microbial polysaccharides gellan and xanthan. BC/PUL biocomposite with prebiotic properties was obtained by cocultivation of *Komagataeibacter xylinus* and *Aureobasidium pullulans*, BC and PUL producers respectively, on molasses medium. The inclusion of PUL in BC is proved gravimetrically by scanning electron microscopy and by Fourier transformed infrared spectroscopy. Cocultivation demonstrated a composite effect on the aggregation and binding of BC fibers, which led to a significant improvement in mechanical properties. The developed approach for “grafting” of prebiotic activity on BC allows preparation of environmentally friendly composites of better quality.

## 1. Introduction

Colonization of the gastrointestinal tract with beneficial bacteria is traditionally a problematic task. Most types of microorganisms used as probiotics survive the passage through the gastrointestinal tract. However, the persistence of these bacteria is not satisfactory, even for probiotic strains isolated from the human intestine. To successfully colonize the gastrointestinal tract, a microorganism must not only absorb nutrients and other growth factors but also outcompete the local microbiota. The application of selectively fermentable prebiotic materials may provide the microbial strain of interest with better survival opportunities and competitive advantage over other gut microbes.

The creation of biocomposites for gastrointestinal applications from natural polymers is one of the intensively developing areas of biotechnology and materials science [1]. As a rule, one of the polymers is used as a matrix or carrier, in which another polymer is included [2]. There are several main strategies and methods for developing new materials with a combination of the properties of individual components, which makes it possible to create composites with targeted activity [3].

The main problem in obtaining biocomposites is to find a biological variant of the composite matrix responsible for fiber binding, load transfer, and protection of the final composite. In this area, studies aimed at the development of composite materials using bacterial cellulose (BC) as a scaffold are becoming increasingly popular [4,5]. Many microorganisms are able to synthesize BC, but the main producer is the bacterium *Komagataeibacter xylinus* [6]. From a chemical point of view, BC is a linear polymer consisting of elementary units of anhydro-d-glucopyranose connected by 1,4-β-glycosidic bonds, and, since it is an exopolysaccharide synthesized by microorganisms, it is a chemically pure extracellular product that does not contain lignin, resins, fats, and waxes [7,8].

In addition to high purity, BC is characterized by excellent crystallinity and high polymerization degree. Equally important are such physical parameters of BC as flexibility, high porosity, various shape, low size, and high water holding [9]. It should be noted that BC is a nontoxic, biodegradable, and biocompatible material, which has led to its widespread use in biomedicine and other related fields [10,11].

Micro- and nanofibrillar structures of BC create a huge potential for constructing various biocomposite materials [12]. The aggregates of BC fibrils occupy an insignificant part of the material’s volume, forming pores of different diameters, which makes it possible to introduce various compounds from nanosized particles to high-molecular-weight polymers into BC [5]. There are also large pores in it, into which cells of prokaryotic and even eukaryotic organisms can be placed. However, special techniques are required to increase the pores sufficient for the incorporation of eukaryotic cells into BC [13]. These technologies are relevant in various areas of tissue engineering, from simple 2-D scaffolds to 3-D printing. As a rule, reinforcing components have biological activity, and since BC is an inert material, they impart new functional properties to the cellulose matrix carrier [14].

There are three main strategies for creating BC biocomposites. The first group—physical modifications (coating, doping, mixing), when both materials retain their unique responsiveness. The second group—chemical modifications (molecular modification, polymer grafting, modification of pore structure). Using these two techniques, it is possible to change the hydrophobicity of the matrix, which is crucial for enhancing cell adhesion during the creation of composites [3]. The third group comprises biological methods, which are often referred to as synthetic approaches to BC composites since, in these methods, the functionalization of BC occurs either during or after its biosynthesis. These methods, in turn, can also be divided into three main types: in situ synthesis, ex situ synthesis, and BC dissolution [3,15,16].

In the in situ strategy, reinforcing materials are added to the culture medium in which the BC producer grows, while the filler is included in its network, i.e., this additive becomes a part of the polymer structure. In ex situ synthesis, the already prepared polymer matrix adsorbs the filler and a composite is obtained.

Thus, modification approaches are associated either with the correction of the structural characteristics of BC during its biosynthesis or with the modification of the material after its synthesis using chemical derivatization. However, for both in situ and ex situ methods, the structure of the BC network is preserved and remains intact throughout the process. In contrast, the BC dissolution method reconstructs its three-dimensional structure. BC is first dissolved in an organic solvent, the reinforcing materials are dispersed in it, and then the BC composite film is regenerated from the solution [3,5].

If a biopolymer synthesized by another microorganism is included in the BC matrix, this product of microbial biosynthesis can be integrated with BC. In that way, the composite can be obtained by cocultivation of producers, creating an artificial symbiotic system [3]. This approach, in our opinion, represents an attractive, cost-effective scheme with fewer pretreatment and purification steps, since the additives are biosynthesized by microorganisms and directly incorporated into the growing BC matrix.

While composites based on cellulose and chemically synthesized (not natural) polymers are commonly referred to as eco- or biocomposites, composites based entirely on natural fibers and biopolymers have been called either “green composites” or “green biocomposites” [17].

One of the interesting options for the “functionalization” of BC in this way is to obtain a biocomposite with prebiotic activity. The most general and modern definition of a prebiotic is “a substrate that is selectively used by microorganisms of the host organism and benefits health” [18]. The most commonly used prebiotics are fructo- and galactooligosaccharides, inulin, and lactulose [19]. However, they can also be polysaccharides that are indigestible in the stomach and small intestine, thus providing selective nutrition for saccharolytic probiotic microorganisms [20]. Information about the prebiotic properties of polysaccharides synthesized by microorganisms—that is, obtained by enzymatic means—is increasingly common [21,22]. For instance, many researcher groups have investigated the effect of xanthan gum (XG) and gellan gum (GG) as prebiotics on the growth and activity of probiotic bacteria [23,24,25,26]. XG is an extracellular heteropolysaccharide produced by the microorganism *Xanthomonas campestris* [27]. GG is an exopolysaccharide secreted by the microorganism *Sphingomonas elodea* [28].

The main purpose of this study is to obtain a biocomposite material by cocultivation of a bacterial cellulose producer and a pullulan producer and to investigate its properties. Pullulan (PUL) is a linear unbranched homopolysaccharide produced by the yeastlike fungus *Aureobasidium pullulans* [29].

It has been reported that PUL fermented by the microbiota can beneficially alter the composition of the intestinal microbiota [30]. In this regard, one of the objectives was to determine the probiotic characteristics of three biosynthetic polysaccharides: XG, GG, and PUL. They were determined by selective fermentation with a selected probiotic strain, *Lactobacillus rhamnosus* GG, whose probiotic effect is confirmed through well-established positive effects on human health [31]. With more than 400 publications and 15 randomized double-blind placebo-controlled studies, this strain of lactobacilli is among the most comprehensively worldwide studied, which naturally sparks increasing interest and trust among medical specialists [32].

Another task of the study is to develop a laboratory protocol for cocultivation of BC and prebiotic producers to increase the yield of BC-based composite material with prebiotic activity. In future studies, this material should be used as a combined polysaccharide matrix for the immobilization of probiotics.

## 2. Materials and Methods

### 2.1. In Vitro Assessment of Prebiotic Properties of Microbial Polysaccharides with Lactobacillus rhamnosus GG

The prebiotic activity of GG, XG, and PUL was investigated by the in vitro method described by Huebner et al. [33] with some modifications. Polysaccharides were added to flasks with carbohydrate-free MRS media at a concentration of 1% (*w/v*). MRS with glucose served as a positive control. MRS without a carbon source was used as a negative control. The bacteriological growth media supplements to prepare MRS media and the polysaccharides were purchased from Veld (Almaty, Kazakhstan).

The strains *Lactobacillus rhamnosus* GG (ATCC^®^ 53103^TM^) and *Escherichia coli* (ATCC^®^ 8739^TM^) were purchased from American Type Culture Collection. Overnight cultures of the probiotic strain *L. rhamnosus* GG and enteric organism *E. coli* were inoculated (10^7^ cells/mL) into culture media flasks (1%, *v/v*) and incubated at 37 °C for 24 h. Samples from each flask were drawn periodically to measure the optical density using a UV-1601 PC spectrophotometer (Shimadzu, Kyoto, Japan) (OD_600_). The number of viable bacteria was also calculated after 24 h of incubation on MRS agar (Hi-Media, Mumbai, India) (for lactobacilli) and nutrient agar (Hi-Media, Mumbai, India) (for *E. coli*) at 37 °C. Changes in the pH of fermentation media were determined using pH meter SevenCompact S220 (Mettler Toledo, Urdorf, Switzerland).

The prebiotic activity score (PAS) was determined using the following Equation (1):

(1) $$\mathrm{PAS}=\frac{Prebiotic\;\left(logP24h-logP0h\right)\;}{Glucose\left(logP24h-logP0h\right)}-\frac{Prebiotic\left(logE24h-logE0h\right)}{Glucose\left(logE24h-logE0h\right)}$$

where log P is the logarithm of growth (CFU/mL) of probiotic bacteria after 24 h and 0 h on prebiotic and glucose; log E is the logarithm of growth (CFU/mL) of *E. coli* after 24 h and 0 h on prebiotic and glucose.

### 2.2. Preparation of BC/PUL Composite

*K. xylinus* C3 and *A. pullulans* C7 strains were isolated at the Biotechnology department, Al-Farabi Kazakh National University; the cultures were deposited in the Republic Collection of Microorganisms (Nur-Sultan, Kazakhstan).

*K. xylinus* inoculum was obtained by transferring a single colony from an agar culture into a 100 mL standard Hestrin–Shramm (HS) broth medium, and then incubated at a temperature of 30 °C for 48 h. The resulting culture was vigorously shaken to release immobilized cells from the synthesized cellulose film, followed by filtration of the suspension through sterile meshes. The cells were then precipitated by centrifugation at a rate of 10,000× *g*. The titer of cells in the inoculum was determined by optical density and adjusted to a cell density of ≈10^7^ cells/mL using a UV-1601 PC spectrophotometer (Shimadzu, Kyoto, Japan).

*A. pullulans* inoculum was obtained by transferring the yeastlike fungus film from an agar culture (1 cm^2^ in size) into a flask containing 25 mL of Chapek Dox medium (Hi-Media, Mumbai, India). The culture was grown for 3 days on an orbital shaker incubator ES-20 (Biosan, Riga, Latvia) (20 rpm) at 25 °C. The microorganism cells were separated from the culture liquid by centrifugation for 15 min at 10,000× *g*. The titer of cells in the inoculum was determined by the optical density and adjusted to a cell density of ≈10^7^ cells/mL using a spectrophotometer.

Inocula of both cultures (1%, *v/v*) were added to the flasks containing 100 mL of media with glucose and molasses. Molasses medium composition (g/L): molasses—20, Na_2_HPO_4_—2.7, KH_2_PO_4_—1, peptone—5, yeast extract—5, citric acid—1.15, ethanol—10, MgSO_4_ × 7H_2_O—0.5, KCl—0.5, FeSO_4_—0.01. Glucose medium composition (g/L): glucose—50, yeast extract—10, peptone—5, citric acid—1.2, Na_2_HPO_4_—2.7, KH_2_PO_4_—5, (NH_4_)_2_SO_4_—5, NaCl—1, MgSO_4_ × 7H_2_O—0.5. Molasses was provided from the domestic sugar factory „Aksu Kant“ (Aksu district, Kazakhstan). All other media supplements were purchased from Veld (Almaty, Kazakhstan).

Cultivation was carried out statically at 30 °C for 7 days. The developed films were first purified by washing with deionized water for 5–7 min. Then, they were treated with 1% (*w/v*) NaOH at 35 °C for 24 h to remove microbe cells and rinsed with deionized water until the pH of the rinsing solution was 6.8–7.2. The resulting samples were dried at 60 °C to constant weight.

### 2.3. Preparation of BC

Inoculum of *K. xylinus* (1%, *v/v*) was added to the flasks containing 100 mL of medium. Cultivation was carried out statically at 30 °C for 7 days. The obtained cellulose samples were removed from bacterial cells according to the method described above and dried at 60 °C to constant weight.

### 2.4. Preparation of PUL

Inoculum of *A. pullulans* (1%, *v/v*) was added to the flasks containing 100 mL of medium. Cultivation was carried out statically at 30 °C for 7 days. The polysaccharide was precipitated with 96% ethanol in a ratio of 2:1. The separation of the precipitated polysaccharide was carried out by centrifugation for 15 min at 10,000× *g*. At the next stage, the sediment was washed with 55% and 96% ethanol and dried twice with ethyl ether at a temperature of 37 °C until the odor was completely removed.

### 2.5. Detection of PUL Content in BC/PUL Composite

Cellulase solution was prepared by dissolving 50 mg/mL cellulase from *Trichoderma* sp. (Sigma-Aldrich, Taufkirchen, Germany) in deionized water. BC/PUL sample was added to cellulase solution at a ratio of 1:10. The mixture was then put in an orbital shaker incubator at 37 °C for 24 h. The precipitation of PUL was carried out according to the method described above. The amount of PUL in BC/PUL was determined gravimetrically.

### 2.6. Characterization of BC/PUL Composite

#### 2.6.1. Fourier Transformed Infrared (FT-IR) Spectroscopy

FTIR spectroscopy was used to identify the chemical structure of the composite material and possible interactions between their components. BC/PUL, BC, and PUL specimens were analyzed with an FT/IR6200 spectrometer (Jasco, Easton, MD, USA) using a resolution of 2 cm^−1^ and 50 scans per spectrum. The samples were initially dried and powdered. Prior to measurement, the sample disks were prepared by mixing polymers with spectrally pure potassium bromide (Sigma-Aldrich, Taufkirchen, Germany) and compressed.

#### 2.6.2. Scanning Electron Microscopy (SEM)

SEM micrographs were obtained using a scanning electron microscope JSM-7800F (Jeol, Tokyo, Japan) at an accelerating voltage of 5 kV. Dried thin slices of samples were mounted on a metal stub and sputter coated with a platinum–palladium alloy (Pt/Pd 80/20). Image J software (version 1.8.0, National Institutes of Health, Bethesda, MD, USA) was used to analyze images and measure fiber diameters and pore sizes. The diameter of at least 100 fibers was determined and averaged.

#### 2.6.3. Mechanical Characterization

The mechanical data—tensile strength (MPa) and elongation at break (%)—were measured by using the Instron bursting machine (model 3365, Norwood, MA, USA) in uniaxial mode. The BC and BC/PUL films were cut into specimens of 10 mm in width and 10 cm in length. The mechanical properties of each sample were the average values determined from five specimens.

### 2.7. Statistical Analysis

Unless otherwise stated, all the experimental groups were assayed in triplicate and presented as mean ± standard deviation. Data were analyzed using a one-way analysis of variance (ANOVA) with the Tukey test. All statistical analyses were performed using SPSS software (version 28.0, IBM Corp., Armonk, NY, USA). Significance was defined as *p <* 0.05.

## 3. Results

### 3.1. The Effect of Microbial Polysaccharides on the Growth of L. rhamnosus GG

**Figure 1:**
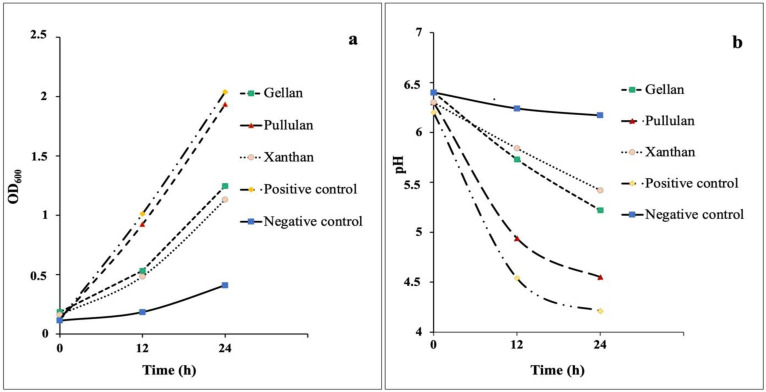
Effects of the media with different added carbon sources on *L. rhamnosus* GG growth (**a**) and the corresponding pH values (**b**).

The biomass concentration of microbial strain was determined by assessing the cell density of MRS media with GG, XG, PUL, glucose (positive control), and without a carbon source (negative control). The cell density of *L. rhamnosus* GG on media with different carbon sources over a 24-h fermentation period is shown in Figure 1a. Carbohydrate-free MRS served as a negative control, whereas MRS with glucose served as a positive control for comparative purposes.

Data on the growth dynamics of *L. rhamnosus* GG showed that the strain was capable of growth on all tested media. *L. rhamnosus* GG demonstrated the highest growth of biomass on the medium with PUL. By 12 h of fermentation, the cell density was 0.923 ± 0.02 and increased to 1.935 ± 0.03 after 24 h. With GG and XG substrates, the growth of biomass rose slightly during the first 12 h due to carbon source fermentation; after 24 h, it reached 1.247 ± 0.02 and 1.132 ± 0.03, respectively.

The medium with glucose had a positive effect on the growth of the strain. It was expected since glucose is a basic nutrient for *L. rhamnosus* GG [34]. For *L. rhamnosus* GG, growth on all studied polysaccharides was less than on glucose; however, PUL was utilized almost at the same level.

*L. rhamnosus* GG growth in MRS medium without any additional carbon source (negative control) was significantly lower (*p <* 0.05) than in the other variants. The growth without any supplement of carbon sources observed in this experiment could be attributed to other ingredients in MRS medium, such as beef and yeast extracts, which contain some carbohydrates and can be used for bacterial growth. Naganuma et al. [35] reported that microorganisms were able to utilize amino acids for growth. This could explain why probiotic could grow in the negative control sample.

The intensity of metabolic processes of *L. rhamnosus* GG was monitored by the pH changing of the medium, which serves as an indicator of the transformation of sugars into organic acids as the end products of metabolism. The data presented in Figure 1b indicate that the change in pH correlated with the intensity of growth and development of bacterial culture within 24 h.

The pH of the fermentation medium containing PUL inoculated with *L. rhamnosus* GG decreased significantly after 24 h of incubation. This suggests that the probiotic can gradually digest and use glucose from PUL, producing acidic compounds that lower the pH. The pH drop of the medium, however, was greater in the positive control compared with that containing PUL. The pH of the negative control remained almost constant during the first 24 h of growth.

GG and XG had little effect on the changes in pH during a 0–12 h fermentation, but decreased by 24 h to 5.22 ± 0.01 and 5.42 ± 0.03, respectively. According to these findings, XG and GG polysaccharides are slow to degrade by the tested microorganisms. In a previous study with xanthan and gellan oligosaccharides, the authors assumed that the branched side chains and β (1 → 4) glycosidic linkages of substrates might explain the delayed degradation [26].

Many microorganisms prefer glucose as an energy source over other carbon sources [36]. Faria et al. [37] reported that XG contained 43% glucose, 32% mannose, and 24% glucuronic acid. GG consists of approximately 60% glucose, 20% glucuronic acid, and 20% rhamnose [38]. It was reported that the composition of monomers and linkage type influence fermentation time, with L rhamnose, arabinose, melezitose, and xylose being fermented slower than D-glucose [39]. This could explain why the microorganism grew slower on the media with the polysaccharides compared to pure glucose.

PUL is comprised of glucose and has linkages that can be hydrolyzed by bacteria. It consists of a linkage pattern with *α* (1 → 4) and *α* (1 → 6) glycosidic bonds. The inhibitory activity of α-glucosidase of *L. rhamnosus* GG strain has been previously reported to vary between 13.5% and 37.9% [40]. This enzyme breaks down the α-1,4-glycosidic and α-1,6-glycosidic bonds, which allows the bacteria to utilize the mentioned carbon source [41].

According to the previous report on the utilization of PUL oligosaccharide as a substrate for *Lactobacillus* strain, enhanced bacterial growth was observed, accompanied by lactic and acetic acid production [26]. Similarly, in the current study, both the growth level of *L. rhamnosus* GG and the metabolic change in pH were statistically significantly (*p* < 0.05) and strongly elevated in PUL samples, indicating its excellent prebiotic quality.

The chemical structure plays a significant role in the process of carbon sources metabolism by lactobacilli, as well as the rate at which it occurs. The fact that the PUL medium was favored for the increase of cellular biomass over other microbial polysaccharide media can be explained by the complex chemical structure of XG and GG. The chemical bonds of XG and GG may have been cleaved more difficult because of the metabolic process of *L. rhamnosus* GG.

The prebiotic activity of substrates was additionally investigated by counting viable cells (log CFU/mL) and determination of prebiotic activity scores.

The distinguishing feature of a prebiotic substrate is that it is selective and cannot be readily fermented by commensal organisms. Therefore, the growth of an enteric bacteria, *E. coli*, was also determined in our study.

The bacterial counts (log_10_ CFU/mL) obtained for *L. rhamnosus* and *E. coli* are compared in Table 1.

The results obtained in this study showed that the probiotic exhibited growth on all tested media; however, the number of log CFU/mL varied depending on the substrate. *L. rhamnosus* GG had a higher growth on medium containing glucose compared with all the tested media. This is due to the fact that carbohydrates with shorter chains are fermented at a higher rate [42]. Supplementation with PUL was found to have a better effect on the viability of probiotic cells compared with media with XG and GG. *E. coli*, similar to many other bacteria, prefers glucose as a carbon source as it promotes faster microorganism growth. Compared with other substrates, the cell count of *E. coli* was very low in the PUL medium (*p <* 0.05), indicating that the probiotic organism’s growth was selectively stimulated.

**Figure 2:**
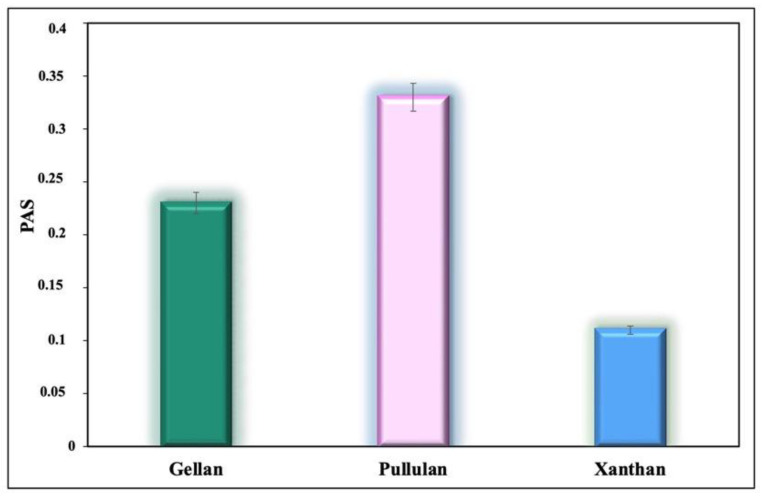
Prebiotic activity score (PAS) of the microbial polysaccharides. Significant difference (*p* < 0.05) was found with different polysaccharides.

Figure 2 shows the PAS values obtained for GG, XG, and PUL.

The PAS values were calculated after 24 h of cultivation. The PAS indicates the ability of a given substrate to stimulate exclusively the growth of a probiotic. Substrates with a positive item of prebiotic activity support good growth of probiotic bacteria, while the number of cells (log CFU/mL) is comparable to the number of cells when grown on a non-prebiotic substrate, such as glucose. However, the growth of *E. coli* grown on prebiotics should theoretically be very low compared with growth on a non-prebiotic substrate. According to Huebner et al. [33], low or even negative values of PAS can be obtained for the microbial strains displaying higher glucose preference over the prebiotic, compared with that of the *E. coli*.

Hence, all microbial polysaccharides tested in our study had a positive effect on the growth of the probiotic strain. The obtained scores decreased in the following order: PUL > GG > XG. PUL demonstrated the highest prebiotic potential since it has the highest PAS-score (0.33 ± 0.01). In other words, PUL appeared to be more compatible as a carbon source to *L. rhamnosus* GG than the other tested polysaccharides. The effect of PUL supplementation on microbial communities observed in this study is consistent with findings from previous works [43,44,45,46,47]. In this regard, the producer of PUL was chosen to obtain a composite material by a synthetic strategy.

### 3.2. Cocultivation of BC and PUL Producers on a Modified Medium with Molasses

The strategy of manufacturing composite materials based on BC by cocultivation of the producing microorganisms has been successfully applied in a number of studies. For instance, the “one-pot” method of cocultivation of *K. хylinus* with other bacteria was successfully applied to the synthesis of BC/hyaluronic acid [48], BC/polyhydroxybutyrate [49], and BC/Nisin [50] composites. This method was also successful when *K. хylinus* was cocultivated with the basidiomycete fungus *Fomitopsis officinalis*, a producer of agaricic acid [51].

**Figure 3:**
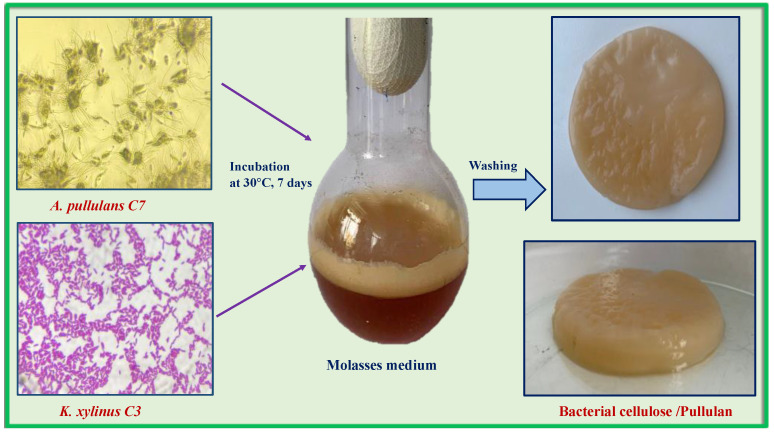
Preparation of bacterial cellulose/pullulan (BC/PUL) biocomposite by cocultivation.

However, the use of artificially created microbial associations may be difficult due to competition for the limiting nutritional component of the medium. An example of this may be the attempt to cocultivate BC and PUL producers, carried out by Hu et al. [52]. It turned out that the yield of the BC/PUL composite (0.249 g/L) was lower than when PUL was directly added to the medium for *K. xylinus* (1.997 g/L). Both polymers are synthesized from glucose. Since the authors used glucose as the carbon source in the medium for the cocultivation of *K. хylinus* and *A. pullulans*, competition for this substrate could well have occurred. In this regard, we tested another medium, previously developed for these producers, where molasses served as a carbon source [53]. It is one of the most cost-effective sources of carbon in the microbiological area. Molasses is a by-product of the final crystallization step in the manufacturing of sugar. It is frequently utilized as a raw material for the preparation of nutritional media because of the substantial amount of sugar it contains. The scheme of the experiment is shown in Figure 3.

According to the data obtained, the yield of BC/PUL composite on the medium with molasses is 1.6 times higher than on the medium with glucose (Table 2).

The effect could occur as a result of the combination of carbohydrates included in molasses (sucrose, glucose, and fructose). Producers first consume glucose before gradually consuming other carbohydrates. It is well-known that cellulose-producing acetic acid bacteria convert glucose to gluconic acid through the process of oxidation, which can cause the pH of the culture broth to drop and limit BC and PUL synthesis [54]. When the producer is grown on molasses, extensive gluconic acid production does not take place and the pH level remains almost at the same level (pH range from 5.3 to 6.2). In addition to sugars, molasses contain many organic nitrogen-containing compounds such as amino acids, nucleic acids, and vitamins in addition to sugars, which can function as growth promoters for the cocultured microorganisms.

In addition, stimulation of the BC film formation can be caused by PUL present in the culture medium. Fang et al. and later Liu et al. reported that the addition of some polysaccharides (such as levan, xylan, XG, and carboxymethylcellulose) to the fermentation medium increases the yield of BC [48,55]. The alleged mechanism is that polysaccharides can bind to precrystallized BC microfibrils, thus interfering the cocrystallization process, which is the rate-limiting step of BC production [56]. During BC synthesis, glucose chains synthetized inside the bacterial cell exit it through tiny pores that are present on its cell membrane. After excretion, parallel adjacent BC protofibrils cocrystallize to form larger microfibrils. BC microfibrils form large ribbons by two main processes: (1) physical aggregation, in which BC microfibrils are joined together due to hydrogen bonds; (2) chemical binding, in which BC microfibrils are linked by polysaccharides such as hemicellulose or carboxymethylcellulose. Cocrystallization, physical aggregation, and chemical binding occur simultaneously, but to varying rates and degrees [55]. The influence of PUL on the formation of BC could take place in all these processes.

**Figure 4:**
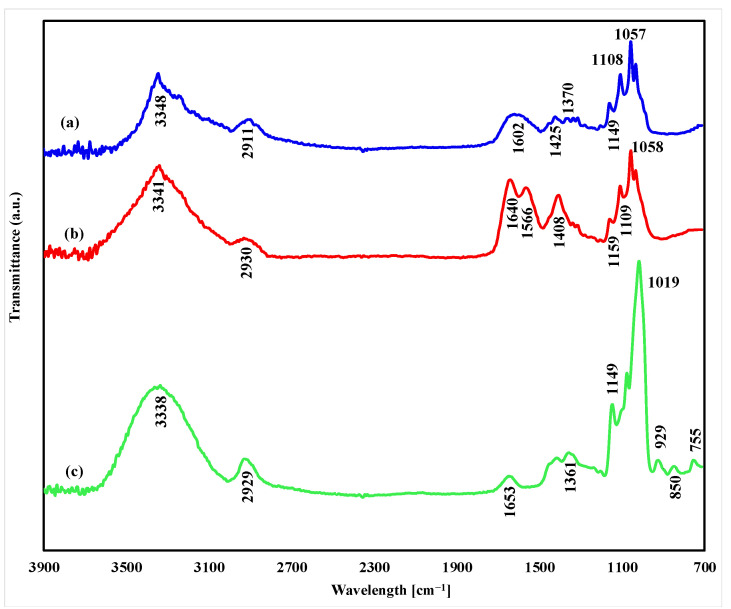
FTIR spectra of (**a**) BC/PUL, (**b**) BC, and (**c**) PUL.

Our results seem to support the data of Hu et al. that PUL interacts with BC and disrupts cocrystallization, stimulating BC production [52]. The proposed reason why the productivity of the film increases is because PUL is incorporated into the BC network matrix during its synthesis, forming a “hybrid” BC/PUL biopolymer. In order to prove this, we used several approaches. Firstly, the synthetized composite was subjected to cleavage by the cellulase enzyme. As the BC dissolved by this treatment, the water-soluble PUL (if it was present in the composite) appeared in the solution from which it was precipitated with ethanol, dried, and weighed. The data obtained in this series of experiments indicate that 37–40% of the synthesized PUL was bound in the cellulose matrix (Table 2, last row). Secondly, FTIR spectral analyses were performed to examine chemical interactions between BC and PUL components. The FTIR spectra of BC, PUL, and BC/PUL are shown in Figure 4.

In the FTIR spectrum of the BC/PUL sample, the characteristic peaks of both components can be found, indicating its composite nature. The peaks at 3348 cm^−1^ and 2911 cm^−1^ exist in all specimens due to the pervasiveness of phenolic compounds containing free hydroxyl groups and C-H stretching vibration, respectively [57]. The absorption peak at 1602 cm^−1^ is related to the stretching vibration of C=O in acryl ketones [58], the stretching vibration of C=C in aromatic rings [59,60], and the bending vibration of O-H bond in phenols [61]. The redshift of the peak in this spectrum compared with the individual BC and PUL spectra can be ascribed to the hydrogen bonding between the polar groups of the components in the BC/PUL sample. The presence of hydrogen bonding sites in the BC contributes to the chemical interaction of cellulose with different polymers [62].

The peaks that appeared at 1425 cm^−1^ and 1370 cm^−1^ are attributed to the bending vibration of C-H bonds [61] in the chemical composition of BC and PUL, respectively. The stretching vibrations of the carbon–oxygen bonds of cellulose are expressed in a set of very well-structured peaks at 1108 cm^−1^ and 1057 cm^−1^. The peak that appeared at 1149 cm^−1^ is the characteristic peak of C-O stretching vibration [63] of the PUL structure.

**Figure 5:**
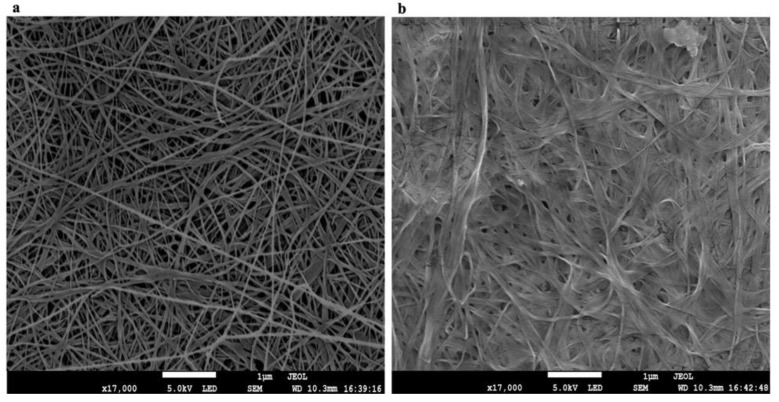
SEM images of BC (**a**) and BC/PUL (**b**) films.

The formation of new bonds between cellulose fibers and PUL can rearrange the microstructure of the BC/PUL film. Structural properties of BC and BC/PUL were examined using a scanning electron microscope. Figure 5 shows fine structure of dehydrated BC and BC/PUL films. The high resolution of the SEM images made it possible to detect differences in the morphology of the surface of the films, as well as to compare the diameter and arrangement of polymer microfibrils relative to each other.

The BC and BC/PUL surfaces exhibit very different micromorphology. In the BC/PUL samples, there was a noticeable associated decrease in the spaces between the fibers (Figure 5b). Nevertheless, the porous structure of the film was generally preserved, displaying numerous macropores and mesopores ranging 20–1000 µm in diameter, as well as nanopores (up to 4 µm). The average measured pore size of the BC/PUL was 61 ± 5 μm, while for BC, it was 103 ± 6 μm, indicating that the PUL content had a significant impact on the pore size of the films. The decrease in the spaces between the fibers is consistent with the data of Gao et al. [56] and Liu et al. [64] on the modification of BC in situ by water-soluble polysaccharides. The average measured diameter of pure BC microfibrils was 63 ± 5 µm, and in the composite—80 ± 4 µm, i.e., they became thicker.

Though the obtained green biocomposites (technically speaking) consist of two major individual components—fibers and a biopolymer matrix—it is the way and degree of binding between both these components that greatly affect the dispersion and mechanical properties of the fibers. For instance, aggregation of PUL molecules with BC can significantly affect the tensile strength of the material, which in turn determines the range of its practical applications.

**Figure 6:**
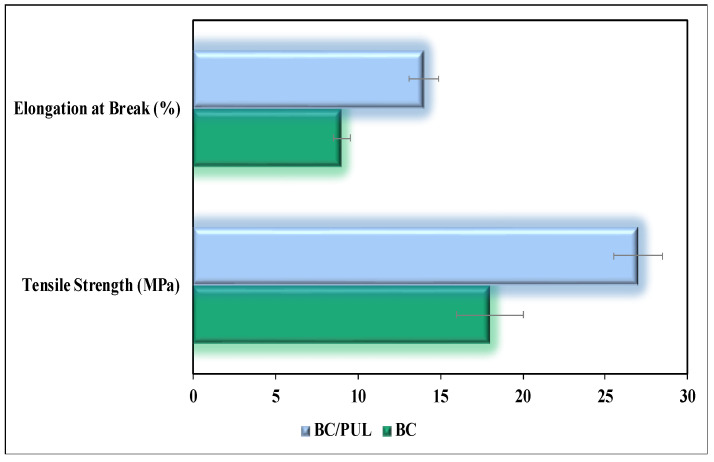
Tensile strength and elongation at break for BC and the BC/PUL composite. All differences were statistically significant (*p <* 0.05).

Since the average thickness of the BC/PUL films was higher than that of pure BC (Table 2), which could affect the mechanical properties, we took samples of the same thickness. Our measurements showed significantly enhanced tensile strength of the BC/PUL composite compared with that of pure BC (Figure 6). In addition, the elongation at break of the composite was 35% greater compared with BC.

The greater the elongation at break, the easier material tolerates impact loads. Thus, PUL had a favorable effect on the mechanical properties of BC, which is consistent with the previously obtained data of Hu et al. [65]. These data are in good agreement with our results, indicating an increase in the number of intermolecular hydrogen bonds in the BC/PUL composite. Indeed, Liu et al. argued that the most important factor affecting the tensile strength is the hydrogen bonds between microfibrils—in particular, between the glucan chains forming them [48]. The binding of PUL to BC microfibrils contributes to their compaction and strengthening due to an increase in the density of hydrogen bonds, which is confirmed by IR spectroscopy.

## 4. Conclusions

This study demonstrated the practical realization of the possibility of cocultivation of *K.* *xylinus* and *A. pullulans* to successfully produce the BC/PUL biocomposite with prebiotic properties. In comparison with other methods, biopolymer production by an artificial symbiotic microbial system has several advantages:

(1) Two or more types of microorganisms are cultivated, stored, and reused together. The growth of all microbial species involved and the production of material components can be achieved under the same conditions. The nutrient medium, temperature, pH, etc. can be kept constant throughout the biosynthetic process.
(2) A biocomposite product can be obtained through a smaller number of pretreatment and purification stages.
(3) Optimization of the fermentation conditions of the coculture system improves the overall production of cellulose film in the presence of PUL in the culture medium. All this makes the biocomposite production technologically more efficient and simpler.
(4) The bio- and green composites obtained via cocultivation of BC- and PUL-producers have improved mechanical properties in terms of tensile strength and elasticity as compared with the individual biopolymers.

The novelty of this research lies in the use of the cost-effective medium in the development of a laboratory protocol for cocultivation of BC and PUL producers to increase the yield of BC-based composite material with prebiotic activity. The developed cellulose composite with prebiotic activity can become a valuable constituent in the production of environmentally friendly composites that combines the fibrillary and matrix phases in a harmonic manner. Although the optimization aspects of cocultivation can be studied more thoroughly, this study is a good start as it has confirmed the potential of cocultivation as a more economical way to produce composite materials in the future.

In future studies, *L. rhamnosus* GG strain, which established the probiotic effect, could be immobilized into the combined polysaccharide matrix to produce a “synergistic synbiotic” to be tested in in vitro and in vivo conditions. It is assumed that such a combination of a prebiotic matrix with a probiotic microorganism could serve as an efficient delivery system for the host organism.

The creation of synbiotic dairy products that have a preventive effect and include probiotic cultures of microorganisms is a topical issue of our time. The synbiotic has a beneficial effect on the host organism by improving the survival of probiotic bacteria in the gastrointestinal tract by selectively stimulating the growth of these bacteria by the prebiotic. The inclusion of a combined polysaccharide matrix with a probiotic strain in the composition of fermented dairy products can provide a high degree of cell viability of probiotic microorganisms during the production, storage, and consumption of such functional foods.

## Figures and Tables

**Table 1:** Bacterial populations grown on different media.

Strain	Substrate	log_10_ CFU/mL		Difference (24 h–0 h)
		0 h	24 h	
*L. rhamnosus* GG	GG	6.03 ± 0.24	7.74 ± 0.28	1.71
	PUL	6.23 ± 0.23	8.02 ± 0.33	1.79
	XG	5.92 ± 0.15	7.54 ± 0.21	1.62
	Glucose	6.44 ± 0.22	8.19 ± 0.34	1.75
*E. coli*	GG	6.22 ± 0.25	7.85 ± 0.29	1.63
	PUL	5.34 ± 0.18	6.85 ± 0.25	1.51
	XG	5.83 ± 0.14	7.63 ± 0.27	1.80
	Glucose	5.74 ± 0.17	7.95 ± 0.28	2.21

GG —gellan gum, PUL—pullulan, XG—xanthan gum; n = 3, groups in different labels have significant differences *p* < 0.05.

**Table 2:** The yield of BC and PUL after 7 days of static cultivation.

	Glucose Medium		Molasses Medium	
	Productivity, g/L	Film Thickness, mm	Productivity, g/L	Film Thickness, mm
*K. xylinus* (BC)	4.6 ± 0.26	4.0 ± 0.21	10.8 ± 0.64	8.0 ± 0.47
*A. pullulans* (PUL)	8.2 ± 0.51	-	10.3 ± 0.54	-
*K. xylinus* + *A. pullulans* (BC/PUL)	10.12 ± 0.62	7.0 ± 0.37	16.8 ± 0.88	12.0 ± 0.67
PUL after BC/PUL digestion with cellulase	3.2 ± 0.18	-	6.7 ± 0.32	-

n = 3, groups in different labels showed significant differences (*p* < 0.05) in all parameters.

## Data Availability

Data are contained within the article.
