# Correction: Zhantlessova et al. Advanced “Green” Prebiotic Composite of Bacterial Cellulose/Pullulan Based on Synthetic Biology-Powered Microbial Coculture Strategy. *Polymers* 2022, *14*, 3224

**DOI:** 10.3390/polym16131802

**Published:** 2024-06-26

**Authors:** Sirina Zhantlessova, Irina Savitskaya, Aida Kistaubayeva, Ludmila Ignatova, Aizhan Talipova, Alexander Pogrebnjak, Ilya Digel

**Affiliations:** 1 Department of Biotechnology, Al-Farabi Kazakh National University, 71 Al-Farabi Avenue, Almaty 050040, Kazakhstan; 2 Department of Nanoelectronics and Surface Modification, Sumy State University, Ryms’koho-Korsakova St. 2, 40000 Sumy, Ukraine; 3 Institute for Bioengineering, Aachen University of Applied Sciences, Heinrich-Mußmann-Straße 1, 52428 Jülich, Germany

## Error in Figure

In the original publication [1], there was an error regarding Figure 5. Specifically, an incorrect figure was used. To address this, a correction has been made to replace Figure 5 with the correct one. The new Figure 5 appears below. The authors state that the scientific conclusions are unaffected. This correction was approved by the Academic Editor. The original publication has also been updated.

## Figures and Tables

**Figure 5:**
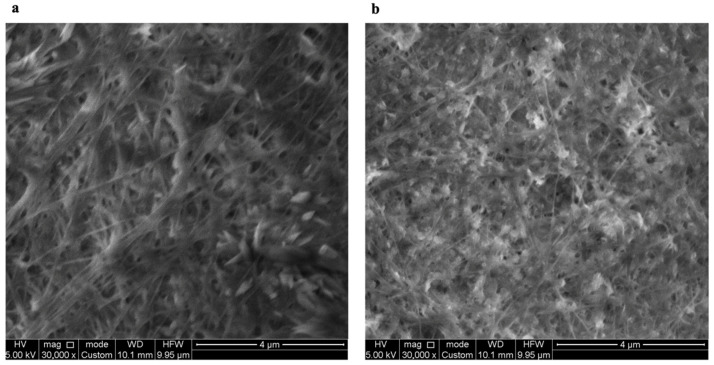
SEM images of BC (**a**) and BC/PUL (**b**) films.
